# Implementation of Listening Visits with Parents of Preterm Infants in an Italian Neonatal Intensive Care Unit

**DOI:** 10.1097/NMC.0000000000001002

**Published:** 2024-04-04

**Authors:** Aurora Scabia, Olena Chorna, Lucia Rocchitelli, Fabrizia Festante, Sabrina Del Secco, Ginevra Costagli, Cristina Riparbelli, Tiziana Controzzi, Cristina Tuoni, Luca Filippi, Andrea Guzzetta

**Affiliations:** **Aurora Scabia** is a Psychologist, Research Trainee, Department of Clinical and Experimental Medicine, University of Pisa, Italy. The author can be reached at a_scabia@hotmail.com; **Olena Chorna** is a PhD Candidate, Department of Neuroscience, Psychology, Drug Research and Child Health NEUROFARBA, University of Florence, Florence, Italy; Department of Developmental Neuroscience IRCCS Stella Maris Foundation, Pisa, Italy.; **Lucia Rocchitelli** is a PhD Candidate, Department of Neuroscience, Psychology, Drug Research and Child Health NEUROFARBA, University of Florence, Florence, Italy; Department of Developmental Neuroscience IRCCS Stella Maris Foundation, Pisa, Italy.; **Fabrizia Festante** is a Post-Doctoral Fellow, Department of Developmental Neuroscience IRCCS Stella Maris Foundation, Pisa, Italy.; **Sabrina Del Secco** is a Clinical Therapist, Department of Developmental Neuroscience IRCCS Stella Maris Foundation, Pisa, Italy.; **Ginevra Costagli** is a Clinical Therapist, Research Trainee, Department of Clinical and Experimental Medicine, University of Pisa, Italy; **Cristina Riparbelli** is Physician at the Neonatology Unit, Azienda Ospedaliero-Universitaria Pisana, Pisa, Italy.; **Tiziana Controzzi** is Physician at the Neonatology Unit, Azienda Ospedaliero-Universitaria Pisana, Pisa, Italy.; **Cristina Tuoni** is Physician at the Neonatology Unit, Azienda Ospedaliero-Universitaria Pisana, Pisa, Italy.; **Luca Filippi** is an Associate Professor, Department of Clinical and Experimental Medicine, University of Pisa, Italy; Neonatology Unit Director, Azienda Ospedaliero-Universitaria Pisana, Pisa, Italy.; **Andrea Guzzetta** is a Full Professor, Department of Clinical and Experimental Medicine, University of Pisa, Italy; Director, Department of Developmental Neuroscience IRCCS Stella Maris Foundation, Pisa, Italy.

**Keywords:** Anxiety, Depression, Feasibility studies, Infant, Intensive care units, Newborn, Parents, Patient-centered care, Premature birth

## Abstract

**Purpose:**

To assess the feasibility of implementing Listening Visits (LV) in an Italian neonatal intensive care unit (NICU).

**Study Design and Methods:**

This feasibility implementation of LV included empathic listening and problem-solving sessions provided by a psychologist to 26 parents of hospitalized preterm newborns. Using the RE-AIM implementation framework, three facets of feasibility were assessed: *reach*, *adoption*, and *implementation*.

**Results:**

It is feasible to integrate LV into the NICU: 76% of families were willing to try LV (*reach*). Listening Visits recipients reported high satisfaction. Twelve of the 16 families (75%) received six or more LV sessions (*adoption*), with mothers attending more sessions. *Implementation fidelity*, defined here as the percentage of LV recipients that received at least four sessions, was 94% among mothers and 30% among fathers.

**Clinical Implications:**

The LV intervention for parental support during the NICU stay is feasible and deemed helpful by parents. Parents were motivated to participate even though their levels of depression, stress, and anxiety were not high. In addition to the use of standardized screening questionnaires, parental requests and clinical team indications should be included in the decision-making for the provision of parental support services.

## Abstract 2

Listening Visits are among a variety of interventions specifically aimed at supporting depressed mothers of hospitalized newborns. In this study, feasibility of implementation of Listening Visits for parents of babies hospitalized in a neonatal intensive care unit in Pisa, Italy included empathic listening and problem-solving sessions provided by a psychologist. Participation by parents was high and they were appreciative of the interactions. Feasibilty of the Listening Visists intervention was established in this clincal setting.

**Figure:**
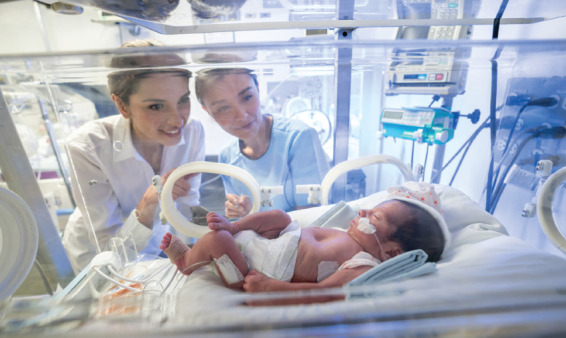
No caption available.

Globally, approximately 14 million or 9.9% of newborns are born preterm, before 37 complete weeks of gestation (Ohuma et al., 2023). A high proportion of these babies are hospitalized in a neonatal intensive care unit (NICU; Walani, 2020). Preterm birth causes a significant amount of psychological distress to the parents, who often report symptoms of depression, post-traumatic stress, and anxiety (Malouf et al., 2022; Staver et al., 2021). Sources of stress include infant appearance and behavior that often conflicts with parental expectations, characteristics of the medical environment, and communication with the health care team (Spinelli et al., 2016). The medical and nursing care environment and procedures of the NICU often deprive parents of fulfilling their expected parental role (Åberg Petersson et al., 2021). This disruption has been shown to be detrimental to parental awareness of their infants' state, needs, and the initiation of their caregiver role (Lundqvist et al., 2019). Evidence highlights some differences in the emotional and psychological effects on mothers and fathers. NICU mothers experience feelings of guilt and isolation, affecting maternal mood and elevated levels of postpartum depression (Lundqvist et al., 2019). Fathers face difficulties in supporting their partner's, family, and employment needs, with an impact on their transition to fatherhood and partner relationship (Åberg Petersson et al., 2021; Mancini, 2023).

In response to the increased awareness of this parental distress, many NICUs have integrated principles of family-centered care (FCC), which shifts the NICU health care team's attention from the premature newborn alone to the premature newborn in the context of their family and community (Mirlashari et al., 2020).

Listening Visits (LV) is among a range of interventions specifically aimed at supporting depressed mothers of hospitalized newborns (Mendelson et al., 2017). This nurse-delivered intervention is comprised of four to six brief sessions based on active listening and collaborative problem solving (Chuffo Siewert et al., 2015; Segre et al., 2013). Important characteristics of LV include that the session focus is decided by the parent and that these sessions often provide a unique opportunity for a NICU parent to discuss their concerns (Segre et al., 2013). US-based evaluations of LV for depressed NICU mothers found that they are associated with a significant decrease of depression and anxiety, and stress levels, with high satisfaction (Segre et al., 2013; Segre et al., 2023).

With significant prior empirical support, the promise of integrating LV as a form of emotional support into the NICU setting is high. Here we report on a study that assessed feasibility of implementing an adapted version of the LV program into an existing FCC program in an Italian NICU. Key adaptations included having a psychologist trained in LV deliver the sessions, making LV available to all NICU parents, including fathers as well as those without elevated depression symptom scores. Although in all prior studies of LV, nurses delivered the intervention, in this feasibility study a multidisciplinary team approach was used and a psychologist provided the LV sessions. This adaptation was necessitated by local norms. Specifically, in the Italian context, the use of a psychologist is required due to the organization of the Public Health Care services. The Italian code of professional ethics, with a specific delineation and limitations of the responsibilities of different professionals, as well as the history of presence of psychologists in Italian NICUs made it most appropriate for a psychologist to implement the LV program.

## Study Design and Methods

### Design, Setting, and Ethics

This prospective feasibility study of LV was carried out between April 2022 and March 2023, in a Level III, 28-bed NICU in Pisa, Italy. Of the 28 beds, 8 are intensive care and 20 are sub-intensive special-care beds. The study was approved by the Tuscan Regional Pediatric Ethical Committee.

### Procedures

**Recruitment and eligibility**. NICU parents were invited to complete a study-specific screening questionnaire to assess their eligibility to enroll in the feasibility trial. Inclusion criteria include having a hospitalized newborn, gestational age 34 weeks or less. Exclusion criteria included having a language barrier (when translation was not possible), having a mental health condition requiring acute psychiatric support, or having severe depression, as indexed by a score of 20 or more on the Edinburgh Postnatal Depression Scale (EPDS; Cox et al., 1987; McCabe-Beane et al., 2016), or a response on item 10 indicating presence of suicidal ideation. A member of the research team, who was not the LV provider, invited eligible parents to receive LV as part of a study. Interested and eligible parents completed informed consent and then the baseline questionnaires.

**Listening Visits**. Following parental consent, the psychologist met with the parents to initiate the study protocol. The LV-session schedule was based on the availability of both parties and around the infant care schedule. The psychologist worked in close collaboration with NICU nursing staff who shared their clinical impressions about the newborn and helped in communications and the organization of sessions in relation to infant care needs. When both parents opted to receive LV, sessions were arranged with each parent individually, unless the couple requested joint session. At the beginning of each session, the parent chose whether to have the LV session at the infant's bedside (in a non-private room), or in a private consultation room.

### Measures

**Demographics**. A study-specific questionnaire assessed parental demographic, social, and family characteristics.

**Parental emotional distress**. The EPDS is a 10-item self-report questionnaire that assesses depressive symptoms in postpartum women (Cox et al., 1987). The Depression Anxiety Stress Scale Short Version (DASS-21) is a 21-item self-report scale that assesses negative psychological states such as depression, anxiety, and stress (Antony et al., 1998). The Perinatal Assessment of Paternal Affectivity scale (PAPA) is an eight-item validated self-report scale that assesses perinatal affective disorders in fathers (Baldoni et al., 2022).

**Satisfaction**. The Client Satisfaction Questionnaire (CSQ) is an eight-item scale that assesses satisfaction with treatment (Larsen et al., 1979). A modified version was used for this study.

### Feasibility Assessment Conceptual Framework

RE-AIM is a conceptual framework used to guide the implementation of evidence-based interventions as well as to assess implementation outcomes (Glasgow et al., 1999). In this study, three elements of RE-AIM assessed the feasibility of implementing LV in an Italian NICU. The first element, *reach*, was defined as the proportion of eligible families who agreed to receive LV, thus indicating their acceptance of the idea. In addition to this initial positive outlook, post-LV satisfaction was also assessed using a modified version of the CSQ. The second RE-AIM element, *(parental) adoption*, was defined here as the percentage of families who completed a full course of LV, i.e., at least six sessions. The third RE-AIM element assessed in this study, *implementation*, is defined as fidelity to the various elements of an intervention. In this study, *implementation (fidelity)* was operationalized as the percentage of LV recipients who received at least a minimal dose of four LV sessions as well as the number of hours dedicated to the intervention by the study psychologist. The latter included the actual time with the parents, as well as the time for session report writing and travel.

## Results

### Participants

Twenty-six parents received LV, representing 16 families, 10 of which included both parents and 6 with mother only. Newborn health characteristics, presence of older siblings, parental demographics, levels of parental depression anxiety levels, and stress at study entry are reported in Table 1.

**TABLE 1.** INFANT AND PARENT CHARACTERISTICS

Infant Characteristics	*N* = 19	
Percent females (*n*)	47% (9)	
Mean gestational age in weeks (SD)	30.16 (3.29)	
Median birthweight in grams (range)	1,230 (500–2,000)	
**Parent Characteristics**	**Mothers *N* = 16**	**Fathers *N* = 10**
Number with previous pregnancies (%)	7/16 (44)	n/a
Number with home within 30-minute drive (%)	3/16 (19)	2/10 (20)
Number using NICU parent guest house (%)	6/16 (38)	0/10 (0)
Number with grandparent assistance (%)	8/16 (50)	7/10 (70)
Educational Level (*n*; %)		
No high school	4/16; (25)	3/10; (30)
High school	2/16; (12.5)	1/10; (10)
Bachelor's degree	4/16; (25)	1/10; (10)
Master's degree or higher	6/16; (37.5)	5/10; (50)
Employment Status (*n*; %)		
Self-employed	0/16; 0	2/10; 20
Employee	11/16; 69	8/10; 80
Unemployed	5/16; 31	0/10; 0
Emotional Well-Being at Study Entry		
Mean EPDS (SD)	8.57 (4.3)	5.77 (4.79)
Mean PAPA (SD)	n/a	7.28 (2.26)
Mean DASS-21 (SD)		
Stress	5.4 (4.24)	3.73 (1.31)
Anxiety	1.8 (2.30)	1.06 (1.72)
Depression	2.93 (0.01)	0.93 (3.16)

### Feasibility Outcomes

Results for all three indices of feasibility within the RE-AIM framework were high (Table 2). Seventy-six percent (16/21) of eligible families were willing to receive LV as part of a study (*Reach*). Satisfaction among LV recipients was high (Table 3). Twelve of the 16 families (75%) received six or more LV sessions (*adoption*). As indicated in Table 2, when considered separately, the adoption rate was higher among mothers (12/16; 75%) than fathers (1/10; 10%). The median number of sessions was notably higher among mothers. Implementation *fidelity*, defined as the percentage of LV recipients who received at least four LV sessions, was 94% among mothers and 30% among fathers. The number of hours dedicated to the program was on average 12: 6 hours to deliver LV and 6 devoted to writing notes and organizing data.

**TABLE 2.** LISTENING VISITS FEASIBILITY OUTCOMES: ADOPTION AND IMPLEMENTATION

Outcome	Mothers	Fathers
**Listening Visits Dose**		
Median number sessions per NICU parent (range)	6 (1–11)	1.5 (1–5)
Mean session duration in minutes (SD)	45 (4.2)	45.3 (5.1)
**Percent of Sessions Completed**		
One session	100	100
Two sessions	100	80
Three sessions	100	60
Four sessions	94	30
Five sessions	81	10
Six sessions	75	10
Seven sessions	19	0
Eight sessions	6	0
Nine sessions	6	0
Ten sessions	6	0
Eleven sessions	6	0
**Primary Focus of Sessions (%)**		
Empathic listening	75	83
Problem solving	25	17
**Location of Sessions (%)**		
Bedside	74	59
Private room	22	24
Online	4	17

## Discussion and Clinical Implications

Results support feasibility of implementing an adapted version of the LV protocol in an Italian NICU with both mothers and fathers. The reach of the program was high, 76% of families agreed to use this support, and reported high satisfaction with this service. Among the parents who agreed to use LV, the rate of adoption was also high. Most of those who agreed to try LV attended all six sessions. Implementation rates were strong, only one of the 16 families attended less than four LV sessions, defined as the minimum dose. Even with these positive outcomes, it is important to highlight that in most cases participation involved mothers, whereas father attendance was at a lower rate (Table 2). Another critical component of feasibility is the cost of implementation of the intervention. We determined that the use of LV required an average of 12 hours per family to complete the full program. This information is important for service providers to define the feasibility of the program in the context of their health service provision framework.

**TABLE 3.** PARENTAL SATISFACTION

Modified Client Satisfaction Questionnaire: Item Theme	Mean Rating (SD)^a^
How appropriate was LV help?	3.53 (0.51)
How competent was LV provider?	3.76 (0.43)
How would you rate the quality of service?	3.46 (0.66)
To what extent has program met your needs?	3.15 (0.55)
How satisfied are you with the amount of help?	3.53 (0.51)
Have LV helped you to deal more effectively with your needs?	3.61 (0.50)
Would you suggest LV to a friend?	3.84 (0.37)
In general, how satisfied are you with LV?	3.69 (0.48)

*N* = 13 families

a Ratings range from “1 = not satisfied” to “4 = extremely satisfied.”

Three aspects of parent participation are noteworthy. First, although parents' clinical symptoms at study entry were generally low, they were nonetheless motivated and interested to receive LV. In prior studies of NICU-based LV, only emotionally distressed mothers were offered the intervention (Segre et al., 2013; Segre et al., 2023). Second, our finding in this implementation suggests that LV support might be seen as beneficial by all parents, not just mothers or just parents identified as emotionally distressed. Most of the LV sessions focused on empathic listening rather than problem solving, suggesting that parents value the time to share their experiences (Table 2). Third, within the framework of LV, it is important to support the family to psychologically contain the set of internal emotional experiences that characterize the parental perceptions of the NICU hospitalization of their infant. We observed that mothers had more opportunities to stay close to their infant/s, and both mothers and fathers preferred to remain at infant bedside during the LV sessions when given the choice of bedside or a private consultation room (74% mothers and 59% fathers). Staying close to their infant and observing (during the sessions) has been reported to help parents to enter in contact with the infant and initiate the process of containment of their psychological distress (Cohen, 2018; Negri, 1998).

From the perspective of the service provider, a psychologist in this study, the LV program appeared to be implementable. For example, when parents were not available for the LV session during the psychologist's working hours, an online visit was offered. This option was frequently chosen by fathers who had little availability during working hours. Although initially considered by the psychologist to be less optimal, the online sessions did not differ from the typical sessions in terms of duration and content. However, despite having the more convenient virtual option, the number of sessions attended by fathers was still low, suggesting that there are barriers other than availability.

### Strengths and Limitations

The conduct of this study in an Italian NICU introduced an innovative model of care to this setting. Among LV studies, it is the first use of LV in Italy, as well as the use of a psychologist to provide the LV session. This is the first study in which LV was offered universally to both mothers and fathers as well as those without elevated depression symptoms. Study limitations include a small, self-selected sample, and lack of parental and newborn outcome data at the time of this report. A larger sample, collection of parental outcomes, and a longer follow-up will be necessary to determine the long-term effect of the program in terms of parental mental health and well-being. In conclusion, our experience confirms parental motivation to take part in the program as well as the feasibility of implementing LV in an Italian NICU.

### Acknowledgment

This work was supported by the Horizon2020 BornToGetThere (Grant No 848201), by Pierfranco e Luisa Mariani Foundation (Rete R-21–122), and by the Italian Ministry of Health (Linea 1 RC-2022 and 5 X 1000 Health Research).

The work was done in collaboration with the nursing and medical staff of the Neonatal Unit of Pisa University Hospital, and in particular Francesca Chesi, Francesca Lorenzoni, Marzia Gentile, Marta Del Pistoia, Patrizia Abeni, Mary Giorgetti, Paola Lazzerini, and Bianca Maria Traina.

## CLINICAL IMPLICATIONS

- Listening Visits provide a feasible option to deliver family-centered compassionate care to NICU parents, many of whom are experiencing emotional distress.
- NICU parents value having a time to discuss their experiences and emotions.
- In cases of non-private room hospitalization of the infant, offering parents a choice for LV session location is important.
- Evaluating parental need for emotional support services may not be limited to standard screening questionnaires.
- Fathers are less available for receiving in-person services.
